# Assessment and Rehabilitation in Cervical Radiculopathy—Efficacy of Structured Program with or Without Neurotrophic Agents

**DOI:** 10.3390/life15111690

**Published:** 2025-10-30

**Authors:** Rodica Magdalena Trăistaru, Kamal Constantin Kamal, Diana Kamal, Diana-Lidia Tache-Codreanu, Adina Maria Kamal

**Affiliations:** 1 Department of Physical Rehabilitation, University of Medicine and Pharmacy Craiova, 200349 Craiova, Romania; 2 Department of Family Medicine, University of Medicine and Pharmacy Craiova, 200349 Craiova, Romania; 3 Department of Physical Rehabilitation, Filantropia Hospital, 200134 Craiova, Romania; 4 Department of Rehabilitation, Colentina University Hospital, 020125 Bucharest, Romania; dianatache@yahoo.com; 5 Department of Internal Medicine, University of Medicine and Pharmacy Craiova, 200349 Craiova, Romania; adina.kamal@umfcv.ro

**Keywords:** chronic cervical radiculopathy, multimodal rehabilitation program, neurotrophic supplementation

## Abstract

Chronic cervical radiculopathy (CR) is a common cause of pain and disability in adults. The primary objective of our study was to evaluate the efficacy of a structured, multimodal rehabilitation program, with or without adjunct neurotrophic supplementation, in chronic cervical radiculopathy; secondarily, this study investigates which patients are most likely to benefit from neurotrophic supplementation and aims to assess effects on pain, cervical mobility, disability, and daily activities to guide individualized care. Patients and Methods: In this prospective, randomized controlled trial, 82 patients with chronic CR were allocated to a study group (SG, *n* = 42) receiving a three-month multimodal rehabilitation program plus daily neurotrophic supplementation (named PHSD) or a control group (CG, *n* = 40) receiving the same rehabilitation alone. Outcome measures included the Visual Analogue Scale (VAS), Neck Disability Index (NDI), cervical mobility indexes (CSI—Chin-Sternum Index; OWI—Occiput-Wall Index; TAI—Tragus-Acromion Index), and Katz ADL (Activity of Daily Living) Index, assessed at baseline and after three months. Results: Both groups showed significant improvements, but the SG demonstrated greater reductions in pain (median VAS change: 8.16 ± 0.72 vs. 5.11 ± 0.70, *p* < 0.001) and disability (mean NDI change: 24.71 ± 5.13 vs. 20.90 ± 4.49, *p* < 0.001). Cervical mobility indexes improved in both groups, with larger gains in the SG (*p* < 0.01), supporting the potential benefits of adding neurotrophic supplementation. Conclusions: A structured multimodal rehabilitation program significantly improves pain, mobility, and disability in chronic CR, while combining it with PHSD may enhance these effects. Further randomized trials are needed to confirm these findings and establish standardized conservative treatment protocols.

## 1. Introduction

Chronic cervical radiculopathy (CR) is a common and debilitating condition characterized by neck pain radiating into the upper limb, accompanied by sensory, motor, and reflex deficits in the affected dermatome or myotome. The annual incidence of CR is estimated at 83 per 100,000 adults, with the highest prevalence in middle-aged and older populations, especially among individuals engaged in occupations involving repetitive cervical movements or prolonged static postures. C6 and C7 nerve roots are most frequently affected, accounting for over 80% of cases [1,2].

The pathogenesis of CR involves both mechanical compression of the nerve root and neuroinflammatory processes triggered by disc herniation, osteophyte formation, or ligamentous hypertrophy. These factors lead to impaired microcirculation, ischemia, and the release of proinflammatory cytokines, contributing to nerve root sensitization, pain persistence, and potential chronicity [3,4,5]. Degenerative changes predominate in older adults, while disc herniation is a more common cause of CR in younger patients [6].

Clinically, CR presents with neck and arm pain, paresthesia, muscle weakness, and reduced deep tendon reflexes in the affected distribution. Diagnosis relies on detailed anamnesis, neurological examination, and confirmation through imaging techniques, especially magnetic resonance imaging (MRI), which provides high-resolution visualization of nerve root compression [7,8]. Functional assessment tools such as the Visual Analogue Scale (VAS) for pain, the Neck Disability Index (NDI), and cervical range of motion tests are commonly used to evaluate symptom severity and monitor treatment response [9].

A useful conceptual framework for managing CR is a three-stage approach: (1) structured, multimodal conservative treatment (education, optimization of medication, physiokinesitherapy/physical therapy, cognitive-behavioral therapy, sleep hygiene, and activity management), (2) targeted minimally invasive interventions when the response to conservative therapy is insufficient (for example, image-guided injections, radiofrequency procedures, selected neuromodulation), and (3) surgical interventions for well-defined indications or persistent therapeutic failure, with multidisciplinary preoperative evaluation [10]. Recent guidelines from the North American Spine Society (NASS) and the European Spine Society recommend conservative treatment as the first-line approach for CR, emphasizing multimodal rehabilitation and patient-centered care before considering surgical options [11,12].

Multimodal rehabilitation, including therapeutic exercise, manual therapy, neural mobilizations, and electrotherapy, has demonstrated significant efficacy in reducing pain and disability in patients with CR. Systematic reviews and meta-analyses highlight that individually tailored exercise programs combined with manual techniques can improve cervical mobility, reduce neuropathic pain components, and enhance functional recovery [13,14,15], potentially avoiding surgical intervention in many cases [11,12]. However, recent systematic reviews have highlighted heterogeneity in protocols and limited high-quality evidence defining optimal conservative strategies [13].

Neurotrophic supplementation with B vitamins, uridine monophosphate, and cytidine has shown promise in enhancing nerve regeneration, reducing neuroinflammation, and improving neuropathic pain in various peripheral neuropathies [14,15]. Despite encouraging data from studies on diabetic neuropathy and carpal tunnel syndrome, evidence on their role as adjuvants in the conservative treatment of CR is scarce.

Beyond the scientific relevance, developing an integrated treatment model that combines standardized rehabilitation with neurotrophic supplementation could provide an accessible, non-invasive, and effective approach with immediate applicability in daily clinical practice. Such a model has the potential to improve patient outcomes, accelerate functional recovery, and reduce the need for surgical interventions or prolonged pharmacological treatments in chronic CR.

Objective: The primary objective was to assess the effectiveness of a structured multimodal rehabilitation program, with or without adjunct neurotrophic supplementation, for chronic cervical radiculopathy in a prospective, randomized controlled design, in order to inform an implementable conservative treatment protocol.

Secondary objectives included identifying patients most likely to benefit from adjunct neurotrophic supplementation and assessing effects on pain, cervical mobility, disability, and daily activities to guide individualized care.

## 2. Patients and Methods

### 2.1. Study Design

This prospective, controlled, interventional study was conducted between 9 June–10 September 2025 in the Department of Physical Medicine and Rehabilitation at Filantropia Hospital, Craiova.

We used a computer-generated allocation sequence (1:1; block size 4; stratified by baseline pain) with allocation concealment via sequentially numbered, opaque, sealed envelopes. Patient willingness was considered only for study participation (consent and general eligibility) and did not influence randomization or group assignment.

The nutraceutical supplement was abbreviated as PHSD to avoid trade names, in line with scientific publishing guidelines.

The baseline clinical and demographic characteristics were comparable between groups, and potential confounders were addressed statistically to reduce selection bias and support internal validity.

Participant Selection and Screening: A total of 110 patients diagnosed with chronic CR persisting for more than three months were initially assessed. After applying the eligibility criteria and excluding patients who required surgical intervention or presented contraindications, 82 patients were enrolled and evaluated at baseline (T1).

**Figure 1:**
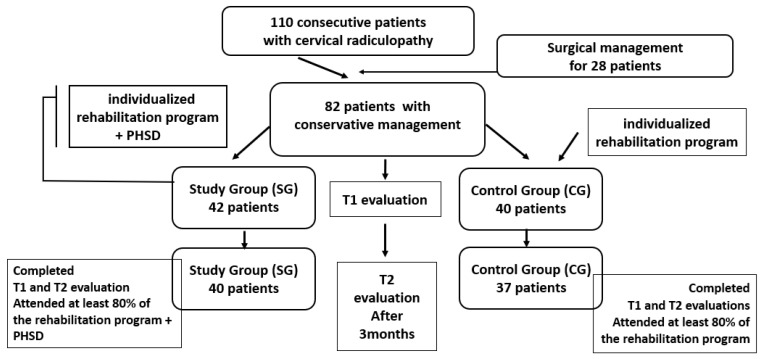
Study flow diagram. PHSD (nutraceutical supplement).

Participants were divided into two groups, as illustrated in the study flow diagram (Figure 1):

- Study Group (SG): This group included 42 patients who followed a standardized physical rehabilitation program complemented by oral administration of PHSD, a neurotrophic supplement, over a 3-month period.
- Control Group (CG): This group included 40 patients who received the same rehabilitation program without PHSD supplementation.

Both groups participated in a 10-session physical rehabilitation program that included electrotherapy, deep oscillation, laser therapy, and an individualized kinesiotherapy protocol. Each session was administered daily over a 2-week inpatient period. Subsequently, patients were instructed to continue the learned exercises at home at least 3 times per week, approximately 45–60 min per session. The SG additionally received PHSD (composition per capsule: uridine, cytidine, B vitamins, and vitamin D3) once daily for 3 months.

Follow-up and Outcome Assessment: Clinical, functional, and inflammatory parameters were assessed at baseline (T1) and post-intervention (T2) by a multidisciplinary team including rehabilitation physicians and physiotherapists. The outcome measures included: cervical alignment indexes (CSI, OWI, TAI), Visual Analogue Scale (VAS) for pain, Neck Disability Index (NDI), Katz Index for daily function, radiographic evaluation of the cervical spine and laboratory markers.

Only patients who completed both evaluations (T1 and T2) and adhered to the intervention protocol were included in the final analysis.

### 2.2. Participants

Eligible patients were identified based on predefined inclusion and exclusion criteria.

Inclusion Criteria: Patients were considered eligible if they fulfilled all the following conditions: (a) age ≥ 50 years; (b) chronic CR symptoms lasting more than 3 months, confirmed by MRI within the previous 6 months; (c) Neck Disability Index (NDI) score > 20% and/or Visual Analogue Scale (VAS) for pain > 4/10 at baseline, indicating clinically significant functional impairment or pain; (d) ability and willingness to participate in and comply with the standardized rehabilitation program and scheduled follow-up visits; (e) stable cardiovascular and respiratory status without uncontrolled or unstable medical conditions; (f) agreement to maintain consistent medication regimens during the study period; (g) absence of other significant upper limb musculoskeletal conditions (e.g., severe osteoarthritis) causing disability; (h) provision of signed informed consent.

Exclusion Criteria: Patients were excluded if they presented any of the following: (a) pregnancy or breastfeeding; (b) requirement for surgical intervention based on clinical and imaging evaluations; (c) intolerance or contraindications to any component of the PHSD supplementation; (d) presence of malignancy, spinal cord disorders, or peripheral neuropathy; (e) signs or diagnosis of cervical myelopathy; (f) laboratory abnormalities defined as liver enzymes (ALT, AST) or urea levels > 2× reference range, or creatinine > 3× reference range; (g) severe psychiatric disorders or cognitive impairment limiting cooperation; (h) active infectious diseases or acute inflammatory conditions at enrollment; (i) active rheumatologic diseases affecting the cervical spine; (j) recent major trauma, burns, or immobilization in the past 6 months; (k) chronic use of medications known to interfere with neuromuscular function, including systemic corticosteroids, sedatives, or neuroleptics; (l) participation in a formal or intensive rehabilitation program within the last 12 months; (m) any other medical condition judged by the investigators to interfere with study participation or safety.

### 2.3. Patients’ Treatment

This study implemented a structured, multimodal rehabilitation program for both SG and CG, specifically designed to alleviate pain, improve cervical spine mobility, and enhance functional independence in patients with chronic cervical radiculopathy. All interventions were carried out under the supervision of specialized physiotherapists.

Program Design and Implementation: The rehabilitation program included conventional physical therapy modalities combined with individualized kinesiotherapy. The SG additionally received daily neurotrophic supplementation with PHSD, whereas the CG received identical rehabilitation interventions without PHSD.

**Components of the Rehabilitation Program (performed in both groups):**

- Electrotherapy (Electrical Muscle Stimulation—Endomed 482, device series 42.400, Enraf-Nonius, Rotterdam, The Netherlands): 30-min daily sessions using biphasic rectangular pulses at 100 Hz, with intensity titrated to visible, pain-free muscle contractions. Electrodes were placed on neck and upper limb muscles to promote neuromuscular activation.
- Low-Level Laser Therapy (ASTAR PhysioGo 500I/501I, Bielsko-Biala, Poland, PhysioGo series): 20-min sessions targeting cervical paravertebral muscles, upper trapezius, and shoulder girdle, using an 808 nm wavelength laser at a power of 100 mW and energy dose of 7 J/cm^2^ per point.
- Deep Oscillation Therapy with manual applicator Personal device (DOP1.1.–INDIVID–Physiomed, device series—2442007, Schnaittach, Germany, Physiomed Elektromedizin AG): 30-min sessions applying high-frequency oscillations (100 Hz) for analgesic and muscle-relaxing effects, followed by low-frequency oscillations (5–25 Hz) to enhance local metabolism. A 5-cm manual applicator was used to treat cervical, trapezius, and shoulder muscles bilaterally. The physiotherapy program was administered daily, 5 days per week, for 2 weeks.

Kinesiotherapy Program. Conducted daily, including the components detailed in Table 1, with progression adapted to individual tolerance and recovery trajectory:

- Warm-Up (5–10 min): neck stretches, shoulder rolls;
- Strengthening Exercises (15 min): isometric neck stabilization, shoulder blade squeezes, upper limb resistance exercises using 0.5–1 kg weights;
- Aerobic Conditioning (15 min): low-intensity walking or stationary cycling to promote circulation and reduce pain sensitivity;
- Mobility and Flexibility Drills (10 min): dynamic neck movements within pain-free range, shoulder girdle mobilizations, upper body stretches;
- Cool-Down (5 min): repetition of stretches and deep breathing exercises for muscle relaxation.

**Table 1:** Kinesiotherapy Program applied to all patients.

Components	Description
Start doing these exercises for 5 repetitions, three times a day. Gradually increase the number of repetitions to about 10 to 20 repetitions. Aims: reduce pain; prepare muscle for reactivation; improve mobility of the shoulders and upper limb: improve grip strength and postural awareness.	
Warm-Up (5–10 min)	Neck stretches. Gentle stretching exercises including neck flexion, extension, lateral flexion, and rotation. Hold each stretch for about 15–20 s. Shoulder rolls. Slowly roll the shoulders forward and backward in a circular motion to reduce tension.
Strengthening Exercises (15 min)	Isometric neck exercises. Press the palm against the forehead and push while resisting with the neck and hold for 5 s. Repeat on each side of the head as well as the back. Shoulder blade squeeze. Sit or stand with arms at the sides and squeeze the shoulder blades together, holding for 5 s. Upper limb strengthening. Sit or stand holding hands on chest with ½ to 1 kg weights in patient’s hands. Alternating arms lift the weights from chest straight up and bring back down. Repeat on each side
Aerobic Conditioning (15 min)	Walking/Stationary cycling. Engage in a light aerobic activity to increase heart rate and blood flow to the muscles, which aids in recovery and pain reduction.
Mobility and Flexibility Drills (10 min)	Dynamic neck movements. Perform controlled neck movements in all directions but within a pain-free range. Shoulder girdle and scapula exercises. Lift shoulders and hold for 1 to 2 s. Then relax the shoulders again. To make this harder, shrug shoulders by keeping both hands on the waist. Move shoulder blades gently back and up (small movement). Hold the contraction for 5 to 10 s. Upper body stretches. Include stretches for the upper back, arms, and chest to improve overall flexibility and reduce muscle imbalances
Cool Down (5 min)	Gentle stretching. Repeat the neck and shoulder stretches from the warm-up to gently relax and lengthen the muscles, especially upper trapezius and scalene. Deep breathing exercises. Focus on slow, deep breaths to relax the entire body and reduce any residual muscle tension.
Education and Self-Management	Posture training. Educate on the importance of good posture to avoid additional nerve compression. Activity modification. Teach how to avoid positions and activities that exacerbate symptoms.

During the entire kinetic program, physiotherapists closely monitored and adjusted exercise intensity and progression according to each patient’s tolerance, ensuring that no pain was provoked during movements. Educational counseling regarding posture correction and activity modifications to prevent exacerbation of symptoms was also provided to all participants.

Specific Intervention for SG: The SG received PHSD. Each capsule contained Uridine-5′-monophosphate (30 mg), Cytidine-5′-monophosphate (30 mg), Cyanocobalamin (5.20 mg), Pyridoxine (1.61 mg), Thiamine (1.27 mg), Vitamin D3 (7.20 mg). PHSD administration was initiated from the first day of the rehabilitation program and continued daily, preferably in the morning with breakfast, to ensure consistent systemic exposure. The timing of PHSD intake was independent of the daily physiotherapy sessions.

Pain Management: All patients received standardized analgesic treatment with paracetamol 1000 mg twice daily for the first 5 days of the rehabilitation program. After this period, treatment with paracetamol was allowed only as rescue medication, and the total consumption was recorded throughout the study to evaluate potential confounding effects.

### 2.4. Parameters and Measurements

An accurate assessment of CR patients is needed to provide adequate management and treatment at an early stage of presentation [16]. A comprehensive evaluation protocol was implemented to assess clinical, functional, and inflammatory parameters, ensuring methodological rigor and supporting the study objectives of evaluating the efficacy of the rehabilitation intervention.

Clinical Parameters:

- Anthropometric measures: Body mass index (BMI) calculated as weight in kilograms divided by height in meters squared (kg/m^2^).
- Cervical mobility indexes: Chin-Sternum Index (CSI): distance between the chin and sternum in maximal cervical flexion; Occiput-Wall Index (OWI): distance between the occiput and wall in maximal cervical extension; Tragus-Acromion Index (TAI): distance measured with the head in neutral position for lateral flexion assessment. Each parameter was recorded in centimeters using a flexible metric tape. These indexes provide quantitative assessment of cervical spine mobility limitations.
- Pain Assessment: Visual Analogue Scale (VAS): patients rated their pain intensity on a 0–10 scale (0: no pain; 10: worst imaginable pain). A minimal clinically important difference (MCID) of 2 points was considered significant improvement, based on validated recommendations for chronic musculoskeletal pain [17].
- Functional Disability Assessment: Neck Disability Index (NDI): a 10-item questionnaire evaluating neck-related disability in daily activities. Each item scored 0–5; total score multiplied by 2 yields percentage disability (0–100%), with higher scores indicating greater impairment. An MCID of 7.5 points and minimally detectable change (MDC) of 10 points were used to interpret clinically relevant change [18,19]. The validated online NDI form was applied to all participants.
- Activities of Daily Living: Katz Index of Independence in Activities of Daily Living (Katz ADL): assesses six basic functions (bathing, dressing, toileting, transferring, continence, feeding), each scored yes/no. Scores: 6 = full function; 4 = moderate impairment; ≤2 = severe functional impairment [20,21,22].

Neurological and Orthopedic Examination: general musculoskeletal inspection; neurological assessment for radicular signs (Spurling test positivity, sensory loss, reflex alterations, myofascial pain syndrome); manual muscle testing of upper limbs and trunk muscles.

Inflammatory Markers: standard screening blood tests including C-reactive protein (CRP) and fibrinogen, measured with automated methods. These markers were measured using commercially available ELISA kits (Biovendor R&D, Brno, Czech Republic), following standardized laboratory protocols, to identify systemic inflammation that could confound chronic pain intensity.

Imaging Studies:

- Magnetic Resonance Imaging (MRI): performed within 6 months prior to inclusion to confirm cervical discopathy and excludes other causes of cervical radiculopathy such as neoplasms or spinal cord compression.
- Radiographic Evaluation: cervical spine radiographs analyzed for spinal alignment, intervertebral space narrowing, osteophytes, vertebral morphology, and calcifications of cervical ligaments.

Outcome Measures Timing: All assessments were conducted at baseline and after completing the 3-month rehabilitation program, allowing objective evaluation of treatment effects over time. Radiographic evaluation of the cervical spine was performed only at T1 to confirm the diagnosis and exclude other structural abnormalities, as significant radiological changes are not expected within a three-month period of conservative treatment, and repeated exposure to radiation was considered unnecessary.

### 2.5. Ethics Approval

The study was conducted with a strong emphasis on safeguarding the safety, dignity, and well-being of all participants, recognizing that individuals with chronic cervical radiculopathy often face substantial pain and disability, which may increase their vulnerability. Participants were thoroughly informed about the study objectives, potential risks and benefits, and the strict measures implemented to ensure confidentiality of personal data, in full compliance with applicable data protection regulations. They were also made aware of their right to withdraw from the study at any time, without the need to provide justification and without any negative consequences for their future care.

Written informed consent was obtained from all participants following comprehensive, clear, and age-appropriate explanations of the procedures, with investigators addressing all patient questions in detail. The study adhered strictly to the ethical principles outlined in the Declaration of Helsinki and followed Good Clinical Practice (GCP) guidelines. Ethical approval for the study was granted by the local independent ethics committee of the Filantropia Hospital, Craiova, under approval number 4665/28, February 2025.

### 2.6. Statistical Analysis

To ensure methodological consistency and transparency, all statistical procedures and parameters applied in this study are detailed below, including the reporting format, sample size considerations, and effect size interpretation.

Statistical analysis was performed using Microsoft Excel (Microsoft Corp., Redmond, WA, USA), SPSS Statistics version 26.0 (IBM Corp., Armonk, NY, USA), and the XLSTAT add-on for Microsoft Excel (Addinsoft SARL, Paris, France). Data were initially recorded in Excel spreadsheets and subsequently imported into SPSS and XLSTAT for advanced analysis and statistical testing.

The distribution of quantitative variables was assessed using the Anderson–Darling and Shapiro–Wilk normality tests. Both tests revealed non-Gaussian distributions for all primary outcome variables (CSI, OWI, TAI, VAS, NDI, KATZ), therefore non-parametric statistical methods were applied throughout. All tests were two-tailed, with a significance level set at *p* < 0.05.

Quantitative variables were reported as mean ± standard deviation (SD) or median with interquartile range (IQR: 25th–75th percentiles), according to the distribution characteristics. Qualitative variables were expressed as absolute and relative frequencies.

For comparisons: within-group differences (T1 vs. T2) were analyzed using the Wilcoxon signed-rank test; between-group differences (SG vs. CG) were assessed with the Mann–Whitney U test;ssociations between continuous variables were explored using Spearman’s rank correlation coefficient (ρ).

Effect sizes were calculated using Cohen’s d, interpreted as follows: small (d = 0.2), medium (d = 0.5), and large (d ≥ 0.8), to enhance the clinical interpretation of statistically significant findings.

Sample size calculation was performed based on the Wilcoxon signed-rank test, assuming a two-tailed α = 0.05 (*Z*α/2 = 1.96), 80% power (*Z*β = 0.84), and an expected effect size of d = 0.8 derived from prior studies evaluating changes in pain and disability scores in cervical spine rehabilitation. The formula used for calculating the sample size considering a finite population was

$$n=\frac{Z^{2}\cdot p\cdot \left(1-p\right)}{E^{2}}\cdot \frac{N}{N+\frac{Z^{2}\cdot p\cdot \left(1-p\right)}{E^{2}}}$$

where *Z* represents the *Z*-score corresponding to the chosen confidence level, *p* the estimated population proportion, *E* the margin of error, and *N* the total population size.

This calculation indicated a required minimum sample size of 26 participants per group to detect meaningful differences. With 42 patients in the SG and 40 in the CG, the final sample provided sufficient power for reliable statistical analysis.

## 3. Results

### 3.1. Baseline Patient Characteristics

A total of 110 patients diagnosed with chronic CR were initially assessed. Following clinical evaluation, 28 patients required surgical treatment and were excluded from the conservative treatment protocol.

Demographic and clinical baseline characteristics were comparable between groups. In both SG and CG, the majority of patients were female, resided in urban areas, and were either overweight or obese according to body mass index (BMI). The average age was 61.71 ± 5.66 years in SG and 60.4 ± 6.31 years in CG, with no statistically significant difference (*p* = 0.3576). A summary of demographic parameters and distribution of socio-professional status is presented in Table 2.

No statistically significant differences were observed between groups regarding gender, residence, occupational status, prior rehabilitation, or BMI category (all *p*-values > 0.05). This homogeneity ensures baseline equivalence and strengthens the internal validity of the study by minimizing confounding factors. Therefore, any post-intervention differences can be more confidently attributed to the addition of PHSD in the SG.

### 3.2. Time-Evolution of Clinical and Functional Parameters in the Study Group

Table 3 presents the evolution of clinical and functional parameters recorded in the study group from baseline (T1) to the final evaluation (T2). All analyzed variables showed statistically significant improvements, confirming the effectiveness of the multidisciplinary rehabilitation intervention. The results of the Wilcoxon signed-rank tests revealed the following:

- Chin-Stern Index (CSI) values decreased significantly from T1 to T2 (*p* = 4.55 × 10^−13^), indicating a reduction in CR pain following the complete therapeutic measures.
- Occiput-Wall Index (OWI) scores improved markedly (*p* = 3.98 × 10^−13^), suggesting better functional integration in occupational activities and reduced disability.
- Tragus-Acromion Index (TAI) scores were significantly reduced (*p* = 4.15 × 10^−13^), reflecting lower fear avoidance behaviors (the patient’s fear of mobilizing the head to avoid exacerbating symptoms) and increased confidence in movement.
- Visual Analogue Scale (VAS) pain scores decreased substantially (*p* = 3.78 × 10^−13^), confirming pain relief after the rehabilitation protocol.
- KATZ Index scores showed significant improvement (*p* = 2.42 × 10^−8^), indicating a better ability to perform activities of daily living independently.
- Neck Disability Index (NDI) scores improved significantly (*p* = 3.17 × 10^−7^), pointing to a decrease in functional limitations associated with cervical spine dysfunction.

**Table 3:** Study Group Parameters at Baseline and Post-Intervention.

Parameters		Mean Value	SD	Min Value	25th Percentile	Median Value	75th Percentile	Max Value
**CSI** **(cm)**	**T1**	4.85	0.84	3	4	5	5	6
	**T2**	2.61	0.58	2	2	3	3	4
**OWI** **(cm)**	**T1**	4.71	0.59	4	4	5	5	6
	**T2**	2.54	0.58	2	2	2.5	3	4
**TAI** **(cm)**	**T1**	15.88	0.73	15	15	16	16	18
	**T2**	13.21	0.84	12	13	13	14	15
**VAS**	**T1**	8.16	0.72	7	8	8	9	9
	**T2**	5.11	0.70	4	5	5	5.75	7
**KATZ**	**T1**	4.26	0.62	3	4	4	5	5
	**T2**	5.76	0.69	4	6	6	6	7
**NDI** **%**	**T1**	24.71	5.13	15	21	25.5	28	33
	**T2**	20.90	4.49	13	17	21	23.75	30

CSI = chin-stern index; OWI = occiput-wall index; TAI = tragus-acromion index, VAS = Visual Analogue Scale; KATZ = Kats scale for activities of daily living, NDI = Neck Disability Index.

**Figure 2:**
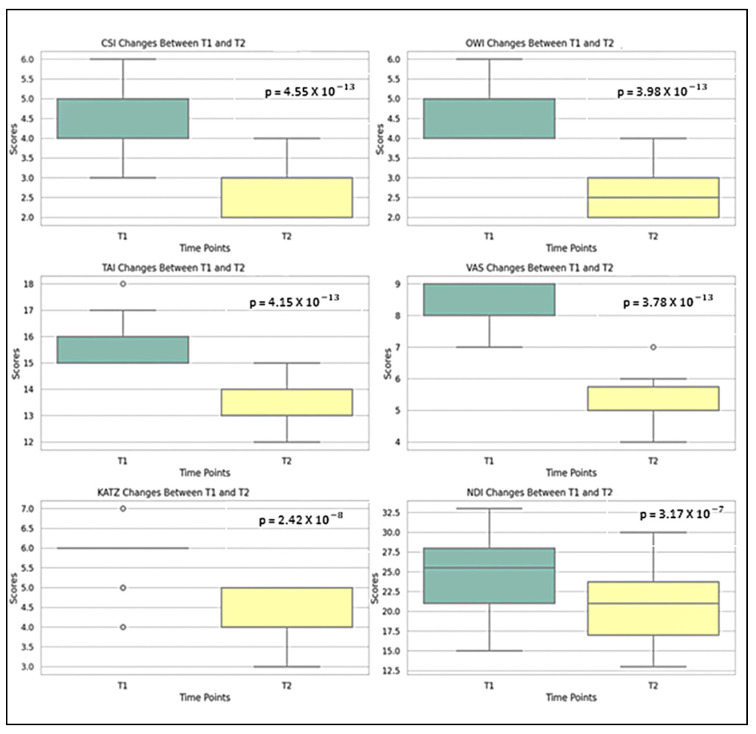
Comparative analysis of T1 vs. T2 parameters in SG (Boxplot Visualization), *p*-values derived from the Wilcoxon signed-rank test.

These findings are illustrated in Figure 2, which highlights the descending trends of pain intensity, and disability, along with improved independence in daily activities.

These results underscore the effectiveness of the rehabilitation program enhanced with PHSD. The consistent direction and magnitude of change across all variables confirm clinically and statistically relevant improvements in pain intensity, posture, cervical mobility, and daily function in the Study Group.

**Figure 3:**
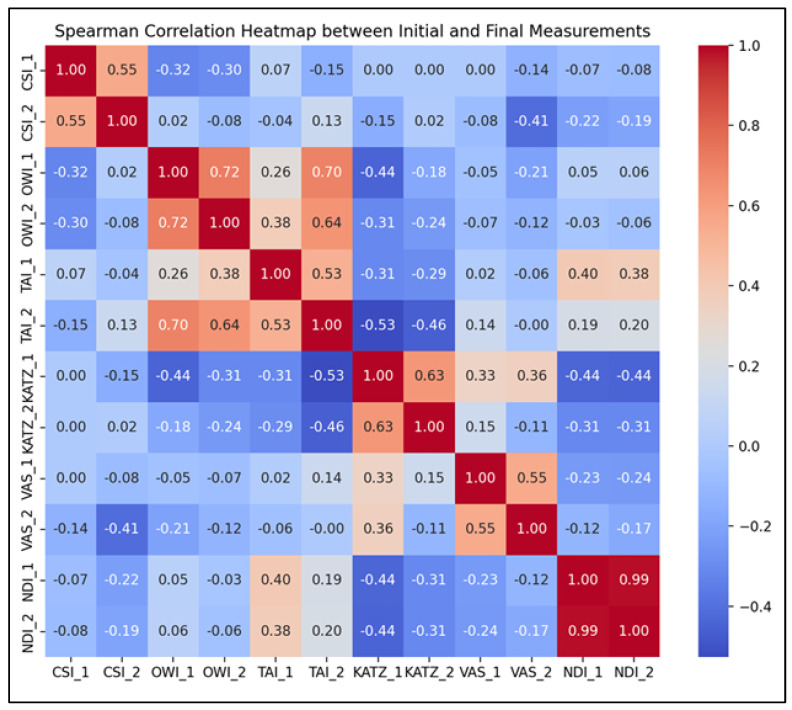
Spearman correlation heatmap for the SG parameters (T1–T2).

To further validate the intervention’s effectiveness, Spearman correlation analyses were conducted to examine the relationships between initial and final values of clinical-functional parameters within the study group (Figure 3). The correlation coefficients and their significance levels are summarized below:

- Chin-Stern Index (CSI): A moderate positive correlation was observed (*r* = 0.55, *p* < 0.001), suggesting that baseline levels of central sensitization were moderately predictive of post-intervention outcomes.
- Occiput-Wall Index (OWI): Displayed a strong positive correlation (*r* = 0.72, *p* < 0.001), indicating a robust link between initial and final levels of perceived occupational functionality and general well-being.
- Tragus-Acromion Index (TAI): Showed a moderate positive correlation (*r* = 0.53, *p* < 0.001), implying consistent therapeutic responsiveness and a predictable reduction in kinesiophobia.
- KATZ Index: Revealed a strong positive correlation (*r* = 0.63, *p* < 0.001), supporting the idea that individuals with greater initial independence retained or improved that status post-intervention.
- Visual Analogue Scale (VAS): Exhibited a moderate positive correlation (*r* = 0.55, *p* < 0.001), suggesting that individuals with higher baseline pain tended to maintain relatively higher levels of discomfort post-treatment, albeit reduced overall.
- Neck Disability Index (NDI): Demonstrated an almost perfect positive correlation (*r* = 0.99, *p* < 0.001), indicating that initial neck disability scores were highly predictive of final scores, potentially reflecting consistent response patterns to targeted cervical rehabilitation.

These strong and statistically significant correlations indicate that baseline assessments of central sensitization, pain, functional status, and disability were highly informative in predicting treatment outcomes. This highlights the clinical relevance of early evaluation and individualized intervention planning, reinforcing the reliability of the selected outcome measures within this rehabilitation framework.

### 3.3. Time-Evolution of Clinical and Functional Parameters in the Control Group

Table 4 provides a detailed overview of the clinical and functional parameters assessed in the control group (CG) at the initial (T1) and final (T2) time points. Although this group did not undergo the PHSD applied to the study group, several statistically significant changes were observed during the follow-up period.

- Chin-Stern Index (CSI): The mean value slightly decreased from 4.00 to 3.90; however, this change was not statistically significant (*p* = 0.346), indicating stability in central sensitization symptoms.
- Occiput-Wall Index (OWI): Showed a modest decrease in the mean score from 4.25 to 4.00, a change that was statistically significant (*p* = 0.033), suggesting some spontaneous improvement in functional well-being.
- Tragus-Acromion Index (TAI): The mean value declined from 15.67 to 13.57, reflecting a significant improvement (*p* = 0.021) in movement-related fear.
- Visual Analogue Scale (VAS): The mean pain score decreased notably from 8.00 to 6.20, with a highly significant change (*p* ≈ 2.55 × 10^−11^), indicating substantial pain relief over time.
- KATZ Index: The average score increased from 5.52 to 5.85, which—contrary to expectations—suggests a deterioration in functional independence (*p* = 0.00031).
- Neck Disability Index (NDI): A small change in the mean value from 23.72% to 23.00% was nonetheless statistically significant (*p* = 0.00011), reflecting a worsening trend in cervical disability.

These results suggest that, despite the absence of an active intervention, measurable changes occurred in the CG. Some parameters (e.g., VAS, TAI) showed favorable evolution, while others (KATZ, NDI) indicated a deterioration of function.

**Figure 4:**
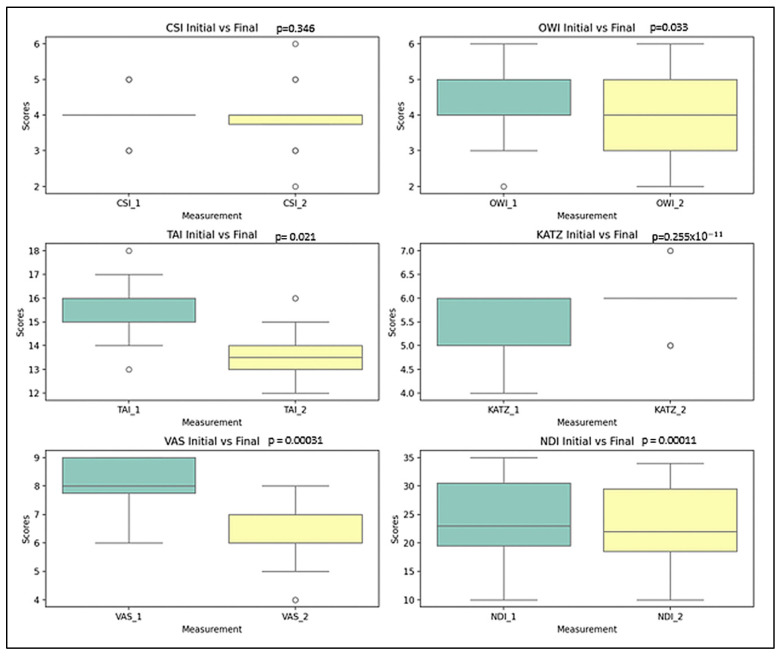
Comparative analysis of T1 vs. T2 parameters in the Control Group. (Boxplot visualization). *p*-values derived from the Wilcoxon signed-rank test.

These statistical findings were visually corroborated by the boxplot representations in Figure 4, which illustrate the distribution and range of scores at both evaluation time points for each parameter. This visualization enhances the understanding of the data’s variability and the clinical significance of the changes observed.

**Figure 5:**
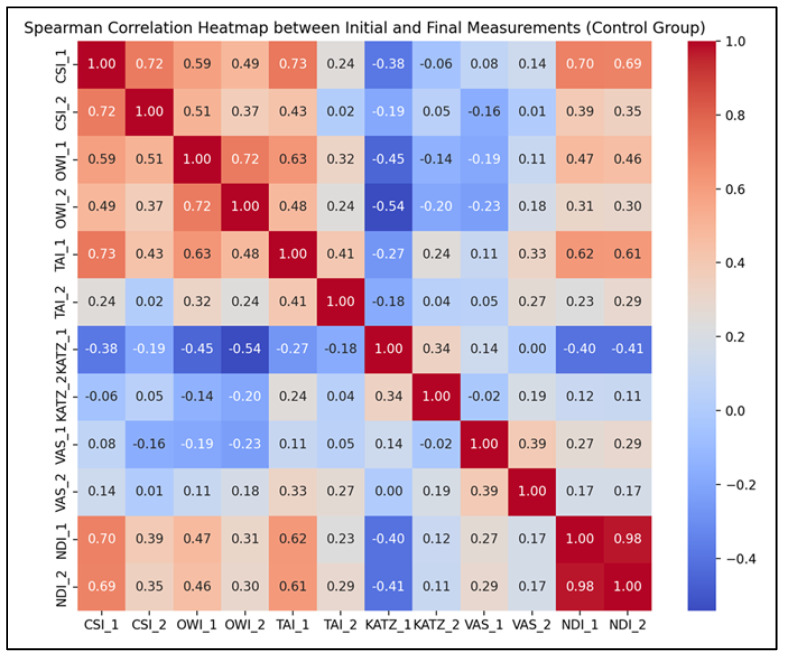
Spearman correlation heatmap for the CG parameters (T1–T2).

In addition to the descriptive and comparative analyses, Spearman correlation tests were employed to evaluate the strength and direction of associations between the initial (T1) and final (T2) scores for each parameter in the control group. The interpretation of correlation coefficients (Figure 5) is as follows:

- Strong positive correlations were observed for CSI (*r* = 0.72, *p* < 0.001), OWI (*r* = 0.72, *p* < 0.001), and NDI (*r* = 0.98, *p* < 0.001), indicating that individuals’ initial values for these parameters were highly predictive of their corresponding final scores, even in the absence of a structured rehabilitation program.
- Moderate positive correlations were recorded for TAI (*r* = 0.41, *p* = 0.0075), VAS (*r* = 0.39, *p* = 0.011), and KATZ (*r* = 0.34, *p* = 0.026), suggesting that although trends were preserved, natural variability influenced the progression of these indicators.

These findings suggest that baseline assessments may offer reliable predictive value for outcome trajectories, even in the absence of structured therapeutic interventions. The results emphasize the clinical importance of conducting comprehensive initial evaluations, which can help anticipate patient evolution over time. Parameters that showed only moderate correlations—such as TAI and KATZ—may reflect domains more vulnerable to behavioral or environmental variability and could particularly benefit from targeted rehabilitation strategies to optimize outcomes.

### 3.4. Study Group Versus Control Group

To assess the comparative impact of the intervention, the Mann–Whitney U test was employed to analyze differences between the study group (SG) and the control group (CG) at both baseline (T1) and post-intervention (T2).

**Baseline Comparison (T1).** At the start of the study, significant intergroup differences were observed for the majority of cervical mobility parameters:

- Cervical Indexes: CSI (*p* = 1.07 × 10^−5^), OWI (*p* = 0.0167), and TAI (*p* = 4.18 × 10^−16^); these results indicate notable disparities in cervical spine alignment and mobility between the two groups prior to any therapeutic intervention.
- Functional Symptom-related parameters: KATZ Index (*p* = 0.598), VAS (*p* = 0.056), and NDI (*p* = 0.360 and 0.791 depending on component); no statistically significant differences were found for these variables, indicating comparable levels of functional independence, pain, and disability at baseline between the groups.

**Post-Intervention Comparison (T2).** Following the 3-month rehabilitation period, statistically significant differences emerged between groups:

- Cervical Indexes: CSI (*p* = 7.92 × 10^−11^), OWI (*p* = 3.67 × 10^−10^), and TAI (*p* = 1.12 × 10^−15^); these highly significant changes favor the study group and support the beneficial impact of the therapeutic intervention on cervical posture and mobility.
- Functional Symptom-related parameters: KATZ Index (*p* = 0.000167) and VAS (*p* = 8.91 × 10^−8^); these outcomes confirm the intervention’s positive effect on daily functioning and pain levels. No significant difference was found at T2 (*p* = 0.162) for NDI, suggesting comparable levels of neck disability across both groups despite improvements in other domains.

**Visual Analysis of Between-Group Differences:**

**Figure 6:**
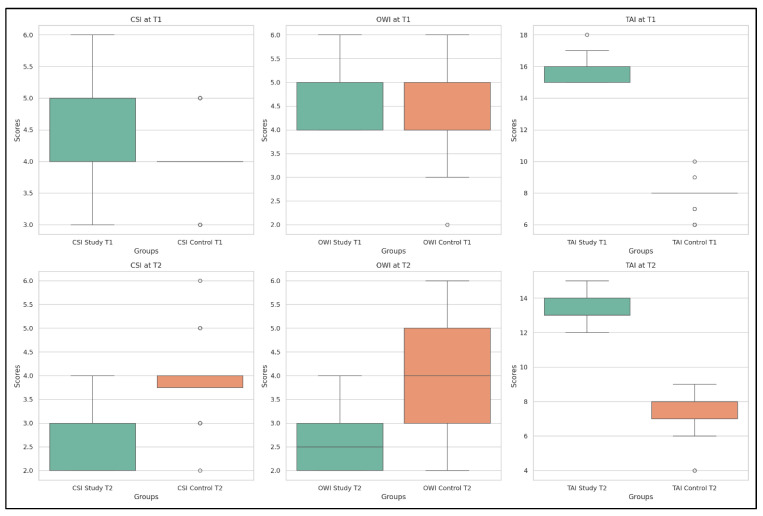
Distributions of CSI, OWI, and TAI values in SG and CG at T1 and T2.

The distributions of the cervical spine indexes—CSI, OWI, and TAI—are illustrated in Figure 6, which provides a clear comparison between SG and CG at both time points.

At T1, the boxplots show overlapping values for CSI and TAI, with minor differences in OWI. At T2, the study group exhibits a marked shift in median and interquartile values for all three indexes, while the control group remains relatively unchanged—highlighting the specific effects of the applied interventions on cervical alignment and postural function.

**Figure 7:**
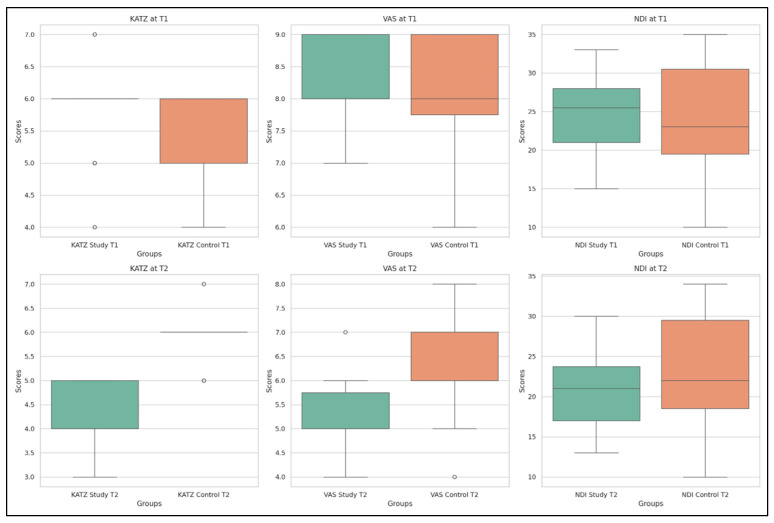
Distributions of KATZ, VAS, and NDI values in SG and CG at T1 and T2.

Additionally, the evolution of functional parameters—KATZ, VAS, and NDI—is displayed in Figure 7, providing visual support for the statistical findings.

The SG shows improved pain scores (VAS) and enhanced functional independence (KATZ) by T2, while the CG maintains a more stable profile. For NDI, both groups show minimal variation, confirming the non-significant intergroup difference in this specific parameter.

## 4. Discussions

This prospective, randomized controlled trial proposed a pragmatic, multimodal approach combining structured physiotherapy and electrotherapy techniques, with or without neurotrophic supplementation, to address chronic cervical radiculopathy (CR).

The results demonstrated that a multimodal rehabilitation program led to improvements in pain, cervical mobility, and disability indices in patients with chronic CR, both in the group receiving neurotrophic supplementation SG and in the CG treated with rehabilitation alone. However, the magnitude of improvement was greater and significant in the SG across all primary outcomes.

The reduction in pain scores observed in both groups underscores the value of comprehensive rehabilitation strategies in managing chronic CR. Our findings align with the meta-analysis by Michaleff et al. (2012), which demonstrated moderate-to-large effects of multimodal physiotherapy interventions on neck pain, and with Thoomes et al. (2012), who reported improvements in pain and disability with conservative treatment [23,24].

These results are consistent with previous clinical guidelines and systematic reviews emphasizing the efficacy of structured physiotherapy, including exercise therapy and manual techniques, in managing mechanical neck disorders and cervical radiculopathy [24,25,26]. Specifically, in our study, the SG achieved a median VAS reduction of 4.5 points—more than twice the MCID of 2 points [17]—while the CG improved by a median of 2.6 points, still exceeding the MCID threshold. These results confirm that a well-structured rehabilitation program alone can substantially alleviate pain in chronic CR over three months, supporting data from prior randomized controlled trials (RCTs) on cervical traction and exercise therapy [27,28].

However, a critical comparison with the literature reveals both strengths and limitations of rehabilitation-only strategies. While intensive physiotherapy has been shown to reduce pain and improve function in CR, systematic reviews highlight inconsistent long-term outcomes, with relapse rates of 20–40% within 6–12 months post-treatment [29]. This inconsistency suggests that rehabilitation alone may not provide durable effects for all patients, particularly those with persistent neuropathic components.

Our data indicate that the addition of PHSD supplementation amplified improvements, especially in pain and cervical mobility indexes (CSI, OWI, TAI), which showed greater normalization in the SG. Disability reduction was also more pronounced in the SG, with a mean NDI decrease of 16.2 points, versus 9.3 points in the CG. While both changes exceed the MCID of 7.5 points for NDI [17], the difference suggests that neurotrophic supplementation may enhance functional recovery beyond what rehabilitation alone can achieve.

These findings parallel observations from clinical trials on uridine and vitamin B complexes in diabetic neuropathy and carpal tunnel syndrome, which demonstrated faster and greater symptom resolution compared with placebo or physical therapy alone [30,31]. The biological rationale is further supported by evidence that neurotrophic agents, such as uridine, acetyl-L-carnitine, alpha-lipoic acid, and B vitamins, can enhance nerve regeneration, modulate neuroinflammation, and improve mitochondrial function, particularly in neuropathic conditions [32,33,34]. Nevertheless, not all literature uniformly supports neurotrophic supplementation. Some authors argue that the placebo effect or improved patient adherence in trials with supplements may partially explain observed benefits [35]. Additionally, meta-analyses on vitamin B treatment for neuropathic pain report high heterogeneity and inconsistent results, particularly in conditions unrelated to metabolic neuropathy [36]. These inconsistencies underscore the need for cautious interpretation of our results and for future randomized studies to confirm the additive effect of PHSD in CR. Importantly, despite these caveats, our findings are biologically plausible. The superior outcomes observed in the SG align with recent evidence emphasizing the role of nucleotides, uridine, and B vitamins in modulating neuroinflammation, promoting myelin repair, and enhancing nerve conduction [37]. These mechanisms may explain why PHSD, when combined with physical therapy, appears to accelerate and augment recovery in chronic CR (a hypothesis warranting further exploration [32,38]). This view is consistent with emerging evidence on the role of neuroinflammation and central sensitization in chronic pain, where immune-glial interactions contribute to persistent hypersensitivity and impaired recovery [39,40,41]. Our findings are further supported by recent studies highlighting the critical link between systemic inflammation and pain sensitivity. For example, Matei et al. demonstrated that elevated inflammatory responses following SARS-CoV-2 infection were associated with significantly reduced pain thresholds in fibromyalgia patients [42]. These observations reinforce the hypothesis that targeting neuroinflammation could be a key strategy to enhance recovery and reduce pain in chronic cervical radiculopathy. Furthermore, our study demonstrates that even in the absence of neurotrophic supplementation, comprehensive rehabilitation can yield meaningful improvements in pain and disability. The CG’s progress highlights the essential role of physiotherapy as a first-line intervention in CR management, supporting guidelines recommending conservative treatment as initial therapy [43].

By directly comparing groups with and without PHSD supplementation in the same rehabilitation framework, our study adds new evidence to the debate over the value of neurotrophic agents in conservative CR treatment. Given that the parameters of physical therapy and kinesiotherapy were similar between groups, we consider it likely that supplementation exerted a genuine neurotrophic effect rather than a placebo effect. The observed additional benefits in the SG suggest that PHSD could be a valuable adjunct in multimodal rehabilitation protocols for patients who exhibit incomplete response to physiotherapy alone or those with marked neuropathic pain features.

Identifying patient profiles with potential for enhanced benefit from neurotrophic supplementation is crucial for optimizing personalized treatment in CR. Our analysis suggests that patients presenting with severe baseline pain (VAS > 6), marked limitations in cervical mobility (OWI or TAI scores > 50%), or high disability indices (NDI > 30) achieved disproportionately greater improvements when neurotrophic supplementation was combined with rehabilitation, compared to those with milder baseline symptoms. These findings indicate that individuals with more pronounced neuropathic features or functional deficits may represent ideal candidates for adjunctive neurotrophic therapy, enabling tailored treatment strategies that maximize recovery across pain intensity, cervical mobility, functional disability, and independence in daily activities. Such stratification could help clinicians prioritize supplementation in patients who are less likely to achieve optimal outcomes through rehabilitation alone, supporting more efficient resource allocation and patient-centered care.

The present findings have clinical implications for the conservative management of chronic CR. While rehabilitation alone proved effective in reducing pain and disability, adding neurotrophic supplementation with PHSD provided additional benefits, with greater and faster improvements in functional outcomes. This combined approach may be particularly valuable for patients with moderate-to-severe CR who are unsuitable for or wish to avoid surgery, offering a non-invasive, safe, and practical alternative. These results are consistent with previous reports showing the effectiveness of active physiotherapy programs in improving neck pain and function [44] and reinforce findings from randomized controlled trials comparing conservative approaches, such as cervical collars or physical therapy, with surgery or wait-and-see strategies [25,45]. Further research is required to identify the patient subgroups who benefit most from this adjuvant strategy, thereby enabling more individualized treatment plans. Stratifying patients based on baseline pain severity, age, or neurofunctional deficits could support this objective. Given the high prevalence and socio-economic burden of chronic neck pain and cervical radiculopathy, strategies that accelerate functional recovery and reduce disability can lower healthcare costs, decrease absenteeism, and improve quality of life [14]. Incorporating neurotrophic agents into multimodal rehabilitation protocols could become a new standard of care, provided that future randomized trials confirm these findings.

Moreover, our structured rehabilitation program, with detailed kinesiotherapy and electrotherapy components, offers a reproducible model for clinicians, and could be integrated into standard physiotherapy protocols for patients with CR.

### Study Limitations

This study presents several limitations that must be acknowledged, although they do not detract from its relevance or scientific contribution.

First, the sample size was relatively small, limiting the power to perform stratified analyses or draw definitive conclusions regarding subgroup responses. Our intention was to simulate real-life clinical practice in rehabilitation medicine, where patient preference and feasibility often dictate intervention pathways. However, as a preliminary clinical investigation, the study offers valuable insights and generates hypotheses for future, larger-scale trials.

Second, the radiological assessment was performed only at baseline, which restricted our ability to monitor structural spinal changes over time. Future protocols should consider incorporating serial imaging to evaluate anatomical correlations with functional outcomes.

Third, analgesic intake was not fully standardized, although rescue medication use was carefully documented. This could have introduced variability in pain perception, yet the multimodal nature of the intervention likely played a more central role in the observed changes.

Fourth, the study was conducted at a single center, which may limit the external generalizability of results. Nevertheless, the standardized treatment protocol and detailed reporting enhance reproducibility in other clinical contexts.

Fifth, the use of a complex multimodal intervention limits the ability to isolate the individual effect of each therapeutic component. While this reflects routine practice in rehabilitation settings, a future factorial or multi-arm design would allow a more granular understanding of treatment mechanisms and guide optimization strategies.

Sixth, no intermediate evaluations were conducted during the 3 months. Although improvements were documented between baseline and final assessments, the precise timing of changes remains unclear. Future studies should incorporate additional timepoints to capture the dynamic evolution of clinical and functional parameters.

Seventh, the multimodal nature of the intervention limits causal attribution at the component level. The comparative design employed (structured program with or without supplementation) was not intended for a component-dismantling or factorial analysis. Consequently, we cannot determine which elements (electrotherapy, laser therapy, deep oscillation, kinesiotherapy, supplementation) predominantly contribute to the observed effects. Future studies should employ factorial designs or optimization frameworks (MOST/SMART) to quantify the contribution of each component and to derive combinations that are both effective and resource-efficient.

Finally, the follow-up duration was limited, which, while sufficient for short-term evaluation, does not inform about the long-term sustainability of treatment effects.

Despite these limitations, this study provides a practically applicable, real-world treatment model for managing chronic cervical radiculopathy using integrated rehabilitation strategies. It offers preliminary but promising evidence on the added value of neurotrophic supplementation, highlights clinically relevant improvements across multiple domains, and underscores the need for individualized, multimodal care approaches.

Future studies should confirm these findings in randomized trials with larger cohorts and longer follow-up to help establish standardized protocols that integrate neurotrophic agents, including nutraceutical supplements, into the conservative management of cervical radiculopathy, thereby supporting the therapeutic potential of comprehensive, patient-centered treatment strategies.

Internal validity was strengthened by strict inclusion and exclusion criteria, homogeneous baseline characteristics, standardized treatment protocols, and consistent assessment procedures performed by the same trained evaluators. However, there were significant between-group differences in some cervical mobility measures at baseline, which may influence the interpretation of post-treatment effects. We conducted analyses adjusted for baseline values and examined change-from-baseline outcomes; however, residual confounding cannot be ruled out.

External validity is supported by the use of widely available rehabilitation techniques and a patient population representative of middle-aged and elderly individuals with chronic CR. Nonetheless, generalization to younger populations, those with acute radiculopathy, or patients with significant comorbidities should be approached with caution.

## 5. Conclusions

Adding neurotrophic supplementation to a structured, multimodal rehabilitation program may yield clinical benefits in chronic cervical radiculopathy.

In this prospective, randomized, controlled trial with balanced baselines, this neurotrophic supplementation produced improvements in pain, cervical mobility/posture, and daily functioning, supporting the utility of an implementable conservative protocol.

However, given the sample size and limited power for subgroup analyses, these findings should be considered preliminary and require confirmation in larger, more robust studies. While within-group results in the SG indicate a clinically relevant effect, robust between-group comparisons are needed to quantify the additional benefit of neurotrophic supplementation over standard multimodal rehabilitation; such evidence can guide treatment personalization and the selection of patients most likely to benefit.

## Figures and Tables

**Table 2:** Baseline demographic and anthropometric characteristics of study participants.

	GENDER		OWN PLACE		PROFESSION (JOB)		PREVIOUS REHABILITATION		TOTAL
	F	M	U	R	A	R	Yes	No	
**SG**	28 66%	14 34%	27 64%	15 36%	20 48%	22 52%	25 60%	17 40%	**42** **100.00%**
**CG**	25 62%	15 38%	25 62%	15 38%	16 40%	24 60%	24 60%	16 40%	**40** **100.00%**
**Total**	53 65%	29 35%	52 63%	30 37%	36 44%	46 56%	49 60%	33 40%	**82** **100.00%**
	*p*-value = 0.6075		*p*-value = 0.3376		*p*-value = 0.6202		*p*-value = 0.6044		
	**Age** **(years)**		**Height** **(meters)**		**Weight** **(kg)**		**BMI** **(kg/m^2^)**		**TOTAL**
**SG**	61.71 ± 5.66		1.66 ± 11.24		81.80 ± 16.94		29.70 ± 5.76		**42**
**CG**	60.4 ± 6.31		1.63 ± 5.97		80.57 ± 13.76		30.02 ± 5.19		**40**
	*p*-value = 0.3576		*p*-value = 0.1366		*p*-value = 0.6693		*p*-value = 0.5905		

SG—study group, CG—control group, F—female, M—male, U—urban, R—rural, BMI—body mass index, A—active, R—retired.

**Table 4:** Control Group Parameters at Initial and Final Assessments.

Parameters		Mean Value	SD	Min Value	25th Percentile	Median Value	75th Percentile	Max Value
**CSI** **(cm)**	**T1**	4	0.67	3	4	4	4	5
	**T2**	3.9	0.74	2	3.75	4	4	6
**OWI** **(cm)**	**T1**	4.25	0.86	2	4	4	5	6
	**T2**	4	0.96	2	3	4	5	6
**TAI** **(cm)**	**T1**	15.67	0.99	13	15	16	16	18
	**T2**	13.57	1.01	12	13	13.5	14	16
**VAS**	**T1**	8	0.72	6	7.75	8	9	9
	**T2**	6.2	0.85	4	6	6	7	8
**KATZ**	**T1**	5.52	0.59	4	5	6	6	6
	**T2**	5.85	0.48	5	6	6	6	7
**NDI** **%**	**T1**	23.72	7.81	10	19.5	23	30.5	35
	**T2**	23	7.66	10	18.5	22	29.5	34

CSI = chin-stern index; OWI = occiput-wall index; TAI = tragus-acromion index, VAS = Visual Analogue Scale; KATZ = Kats scale for activies daily living, NDI = Neck Disability Index.

## Data Availability

Due to confidentiality and regulatory requirements (e.g., GDPR) imposed by the ethics committee, individual participant data cannot be made publicly available.
